# Supplementary material for: Immune Cell Distributions in the Blood of Healthy Individuals at High Genetic Risk of Parkinson’s Disease
Source: Int J Mol Sci. 2024 Dec 20;25(24):13655. doi: 10.3390/ijms252413655 (PMC11728367; doi:10.3390/ijms252413655)

Supplementary Figure S1. Descriptive distribution plots of the Berlin Aging Study II

A. Age distribution in the Berlin Aging Study II

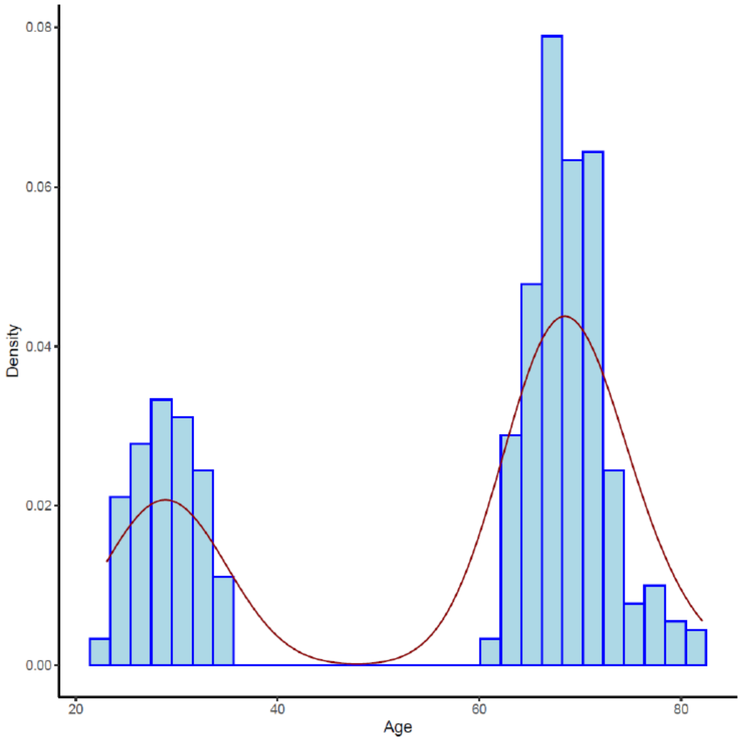

B. Distribution of the polygenic risk score

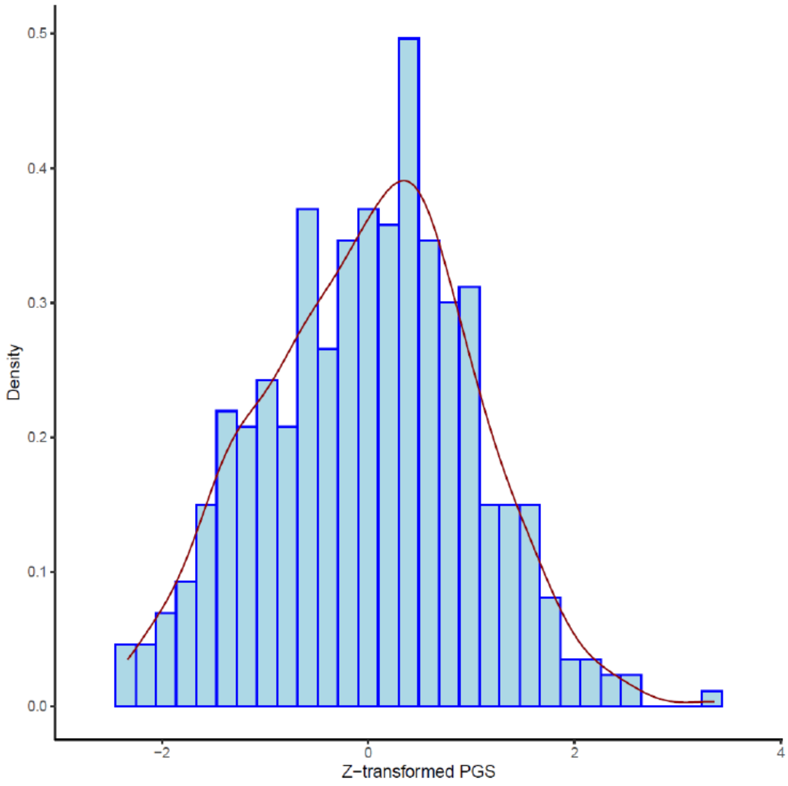

Supplementary Figure S2. Immune cell type distributions in BASE-II before and after transformation and outlier removal

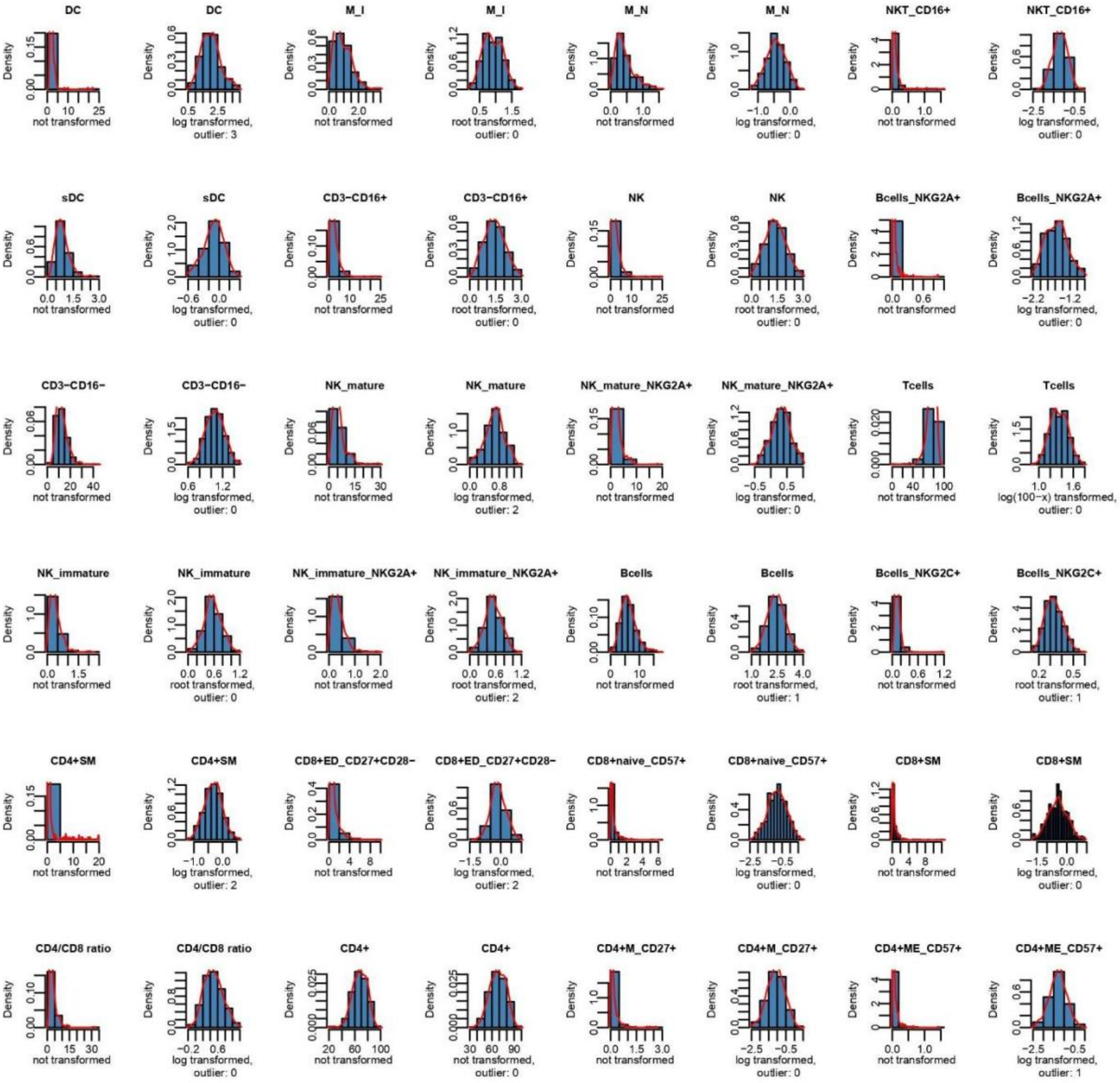

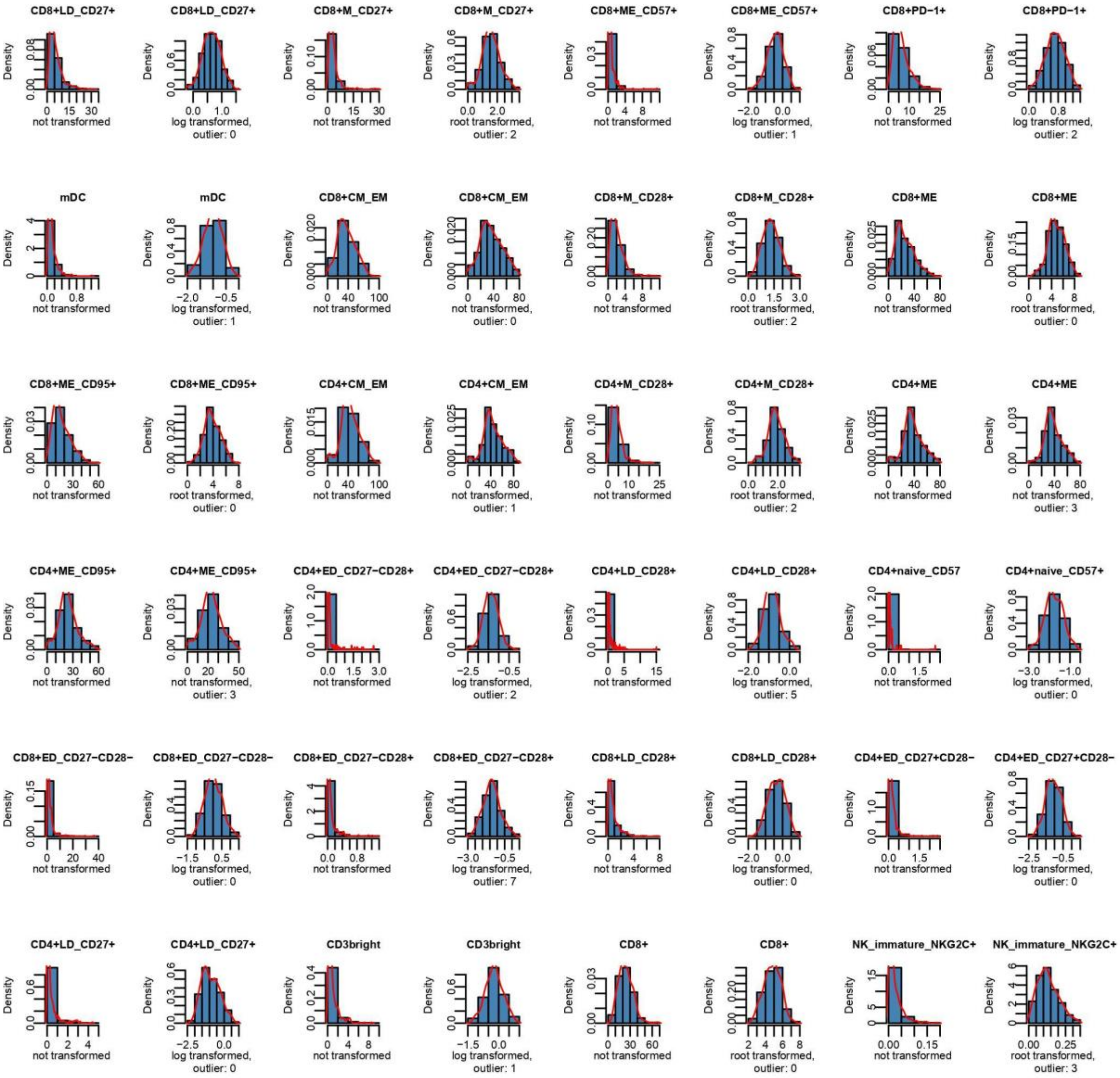
CD8+LD_CD27+
not transformed
CD8+LD_CD27+
log transformed, outlier: 0
CD8+M_CD27+
not transformed
CD8+M_CD27+
root transformed, outlier: 2
CD8+ME_CD57+
not transformed
CD8+ME_CD57+
log transformed, outlier: 1
CD8+PD−1+
not transformed
CD8+PD−1+
log transformed, outlier: 2
mDC
not transformed
mDC
log transformed, outlier: 1
CD8+CM_EM
not transformed
CD8+CM_EM
root transformed, outlier: 0
CD8+M_CD28+
not transformed
CD8+M_CD28+
root transformed, outlier: 2
CD8+ME
not transformed
CD8+ME
root transformed, outlier: 0
CD8+ME_CD95+
not transformed
CD8+ME_CD95+
root transformed, outlier: 0
CD4+CM_EM
not transformed
CD4+CM_EM
root transformed, outlier: 1
CD4+M_CD28+
not transformed
CD4+M_CD28+
root transformed, outlier: 2
CD4+ME
not transformed
CD4+ME
root transformed, outlier: 3
CD4+ME_CD95+
not transformed
CD4+ME_CD95+
not transformed, outlier: 3
CD4+ED_CD27−CD28+
not transformed
CD4+ED_CD27−CD28+
log transformed, outlier: 2
CD4+LD_CD28+
not transformed
CD4+LD_CD28+
log transformed, outlier: 5
CD4+naive_CD57
not transformed
CD4+naive_CD57+
log transformed, outlier: 0
CD8+ED_CD27−CD28−
not transformed
CD8+ED_CD27−CD28−
log transformed, outlier: 0
CD8+ED_CD27−CD28+
not transformed
CD8+ED_CD27−CD28+
log transformed, outlier: 7
CD8+LD_CD28+
not transformed
CD8+LD_CD28+
log transformed, outlier: 0
CD4+ED_CD27+CD28−
not transformed
CD4+ED_CD27+CD28−
log transformed, outlier: 0
CD4+LD_CD27+
not transformed
CD4+LD_CD27+
log transformed, outlier: 0
CD3bright
not transformed
CD3bright
log transformed, outlier: 1
CD8+
not transformed
CD8+
root transformed, outlier: 0
NK_immature_NKG2C+
not transformed
NK_immature_NKG2C+
root transformed, outlier: 3
Density

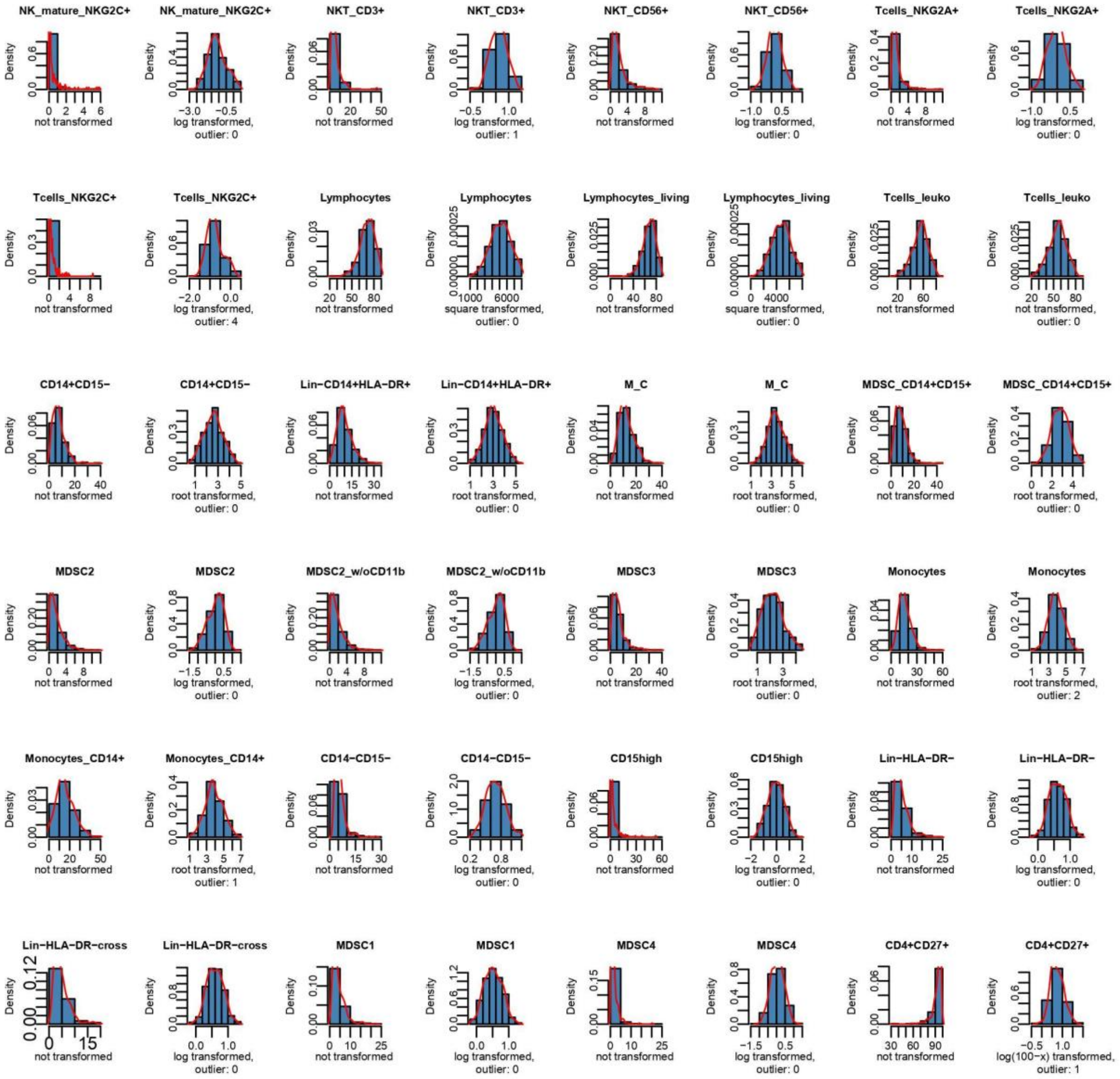
NK_mature_NKG2C+
not transformed
NK_mature_NKG2C+
log transformed, outlier: 0
NKT_CD3+
not transformed
NKT_CD3+
log transformed, outlier: 1
NKT_CD56+
not transformed
NKT_CD56+
log transformed, outlier: 0
Tcells_NKG2A+
not transformed
Tcells_NKG2A+
log transformed, outlier: 0
Tcells_NKG2C+
not transformed
Tcells_NKG2C+
log transformed, outlier: 4
Lymphocytes
not transformed
Lymphocytes
square transformed, outlier: 0
Lymphocytes_living
not transformed
Lymphocytes_living
square transformed, outlier: 0
Tcells_leuko
not transformed
Tcells_leuko
not transformed, outlier: 0
CD14+CD15−
not transformed
CD14+CD15−
root transformed, outlier: 0
Lin−CD14+HLA−DR+
not transformed
Lin−CD14+HLA−DR+
root transformed, outlier: 0
M_C
not transformed
M_C
root transformed, outlier: 0
MDSC_CD14+CD15+
not transformed
MDSC_CD14+CD15+
root transformed, outlier: 0
MDSC2
not transformed
MDSC2
log transformed, outlier: 0
MDSC2_w/oCD11b
not transformed
MDSC2_w/oCD11b
log transformed, outlier: 0
MDSC3
not transformed
MDSC3
root transformed, outlier: 0
Monocytes
not transformed
Monocytes
root transformed, outlier: 2
Monocytes_CD14+
not transformed
Monocytes_CD14+
root transformed, outlier: 1
CD14−CD15−
not transformed
CD14−CD15−
log transformed, outlier: 0
CD15high
not transformed
CD15high
log transformed, outlier: 0
Lin−HLA−DR−
not transformed
Lin−HLA−DR−
log transformed, outlier: 0
Lin−HLA−DR−cross
not transformed
Lin−HLA−DR−cross
log transformed, outlier: 0
MDSC1
not transformed
MDSC1
log transformed, outlier: 0
MDSC4
not transformed
MDSC4
log transformed, outlier: 0
CD4+CD27+
not transformed
CD4+CD27+
log(100−x) transformed, outlier: 1
Density

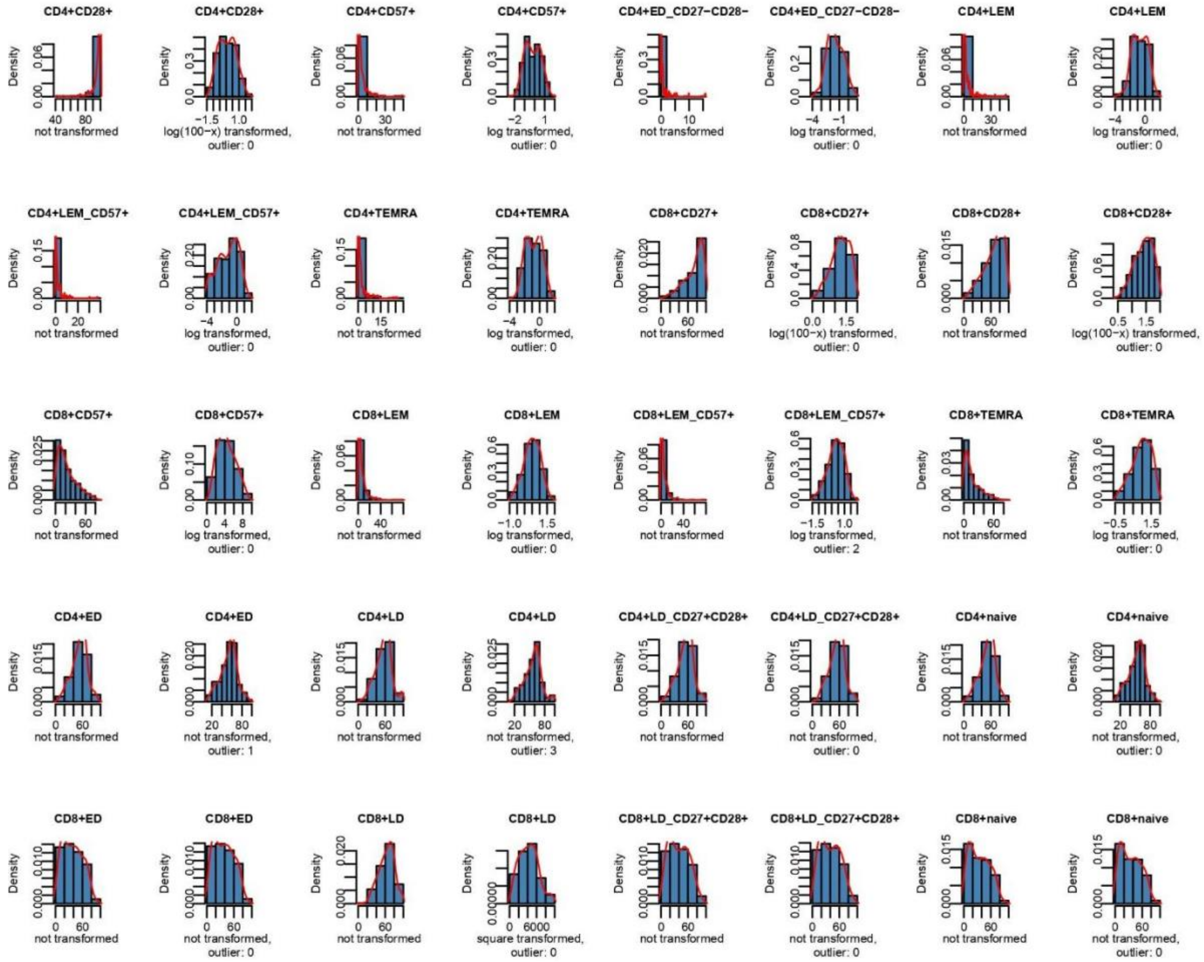
CD4+CD28+
not transformed
CD4+CD28+
log(100−x) transformed, outlier: 0
CD4+CD57+
not transformed
CD4+CD57+
log transformed, outlier: 0
CD4+ED_CD27−CD28−
not transformed
CD4+ED_CD27−CD28−
log transformed, outlier: 0
CD4+LEM
not transformed
CD4+LEM
log transformed, outlier: 0
CD4+LEM_CD57+
not transformed
CD4+LEM_CD57+
log transformed, outlier: 0
CD4+TEMRA
not transformed
CD4+TEMRA
log transformed, outlier: 0
CD8+CD27+
not transformed
CD8+CD27+
log(100−x) transformed, outlier: 0
CD8+CD28+
not transformed
CD8+CD28+
log(100−x) transformed, outlier: 0
CD8+CD57+
not transformed
CD8+CD57+
log transformed, outlier: 0
CD8+LEM
not transformed
CD8+LEM
log transformed, outlier: 0
CD8+LEM_CD57+
not transformed
CD8+LEM_CD57+
log transformed, outlier: 2
CD8+TEMRA
not transformed
CD8+TEMRA
log transformed, outlier: 0
CD4+ED
not transformed
CD4+ED
not transformed, outlier: 1
CD4+LD
not transformed
CD4+LD
not transformed, outlier: 3
CD4+LD_CD27+CD28+
not transformed
CD4+LD_CD27+CD28+
not transformed, outlier: 0
CD4+naive
not transformed
CD4+naive
not transformed, outlier: 0
CD8+ED
not transformed
CD8+ED
not transformed, outlier: 0
CD8+LD
not transformed
CD8+LD
square transformed, outlier: 0
CD8+LD_CD27+CD28+
not transformed
CD8+LD_CD27+CD28+
not transformed, outlier: 0
CD8+naive
not transformed
CD8+naive
not transformed, outlier: 0

Supplementary Figure S3. Heatmap showing the correlations between the corresponding nominally significant cell types per disease

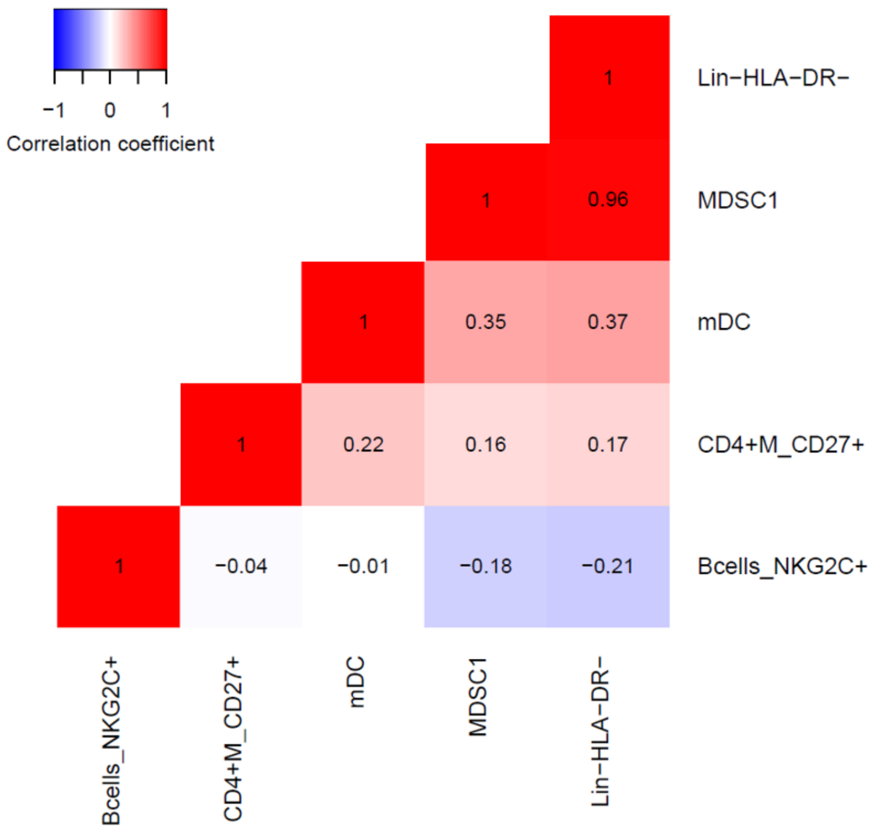

Supplementary Figure S4. Distribution of the residuals of the nominally significant regression analyses of the Parkinson's disease polygenic risk score and the corresponding immune cell proportion in blood

NKG2C+ B cells

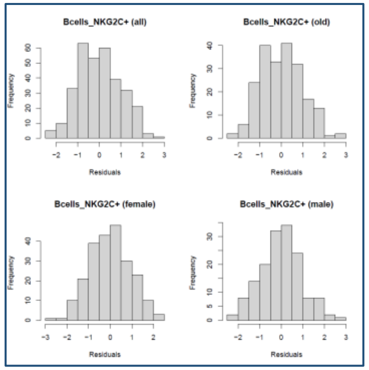

Myeloid dendritic cells

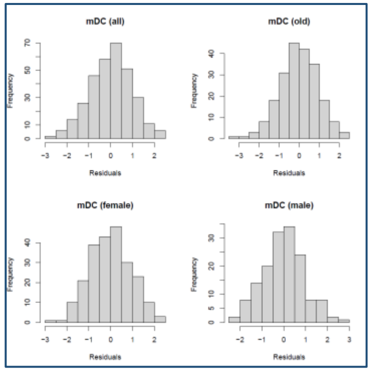

CD27+ CD4+ memory T cells

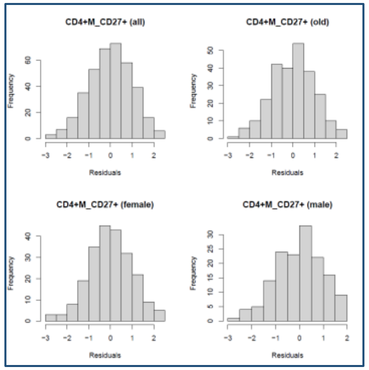

MDSC1

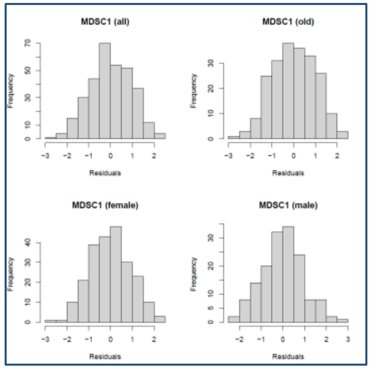

Lin-HLA-DR-

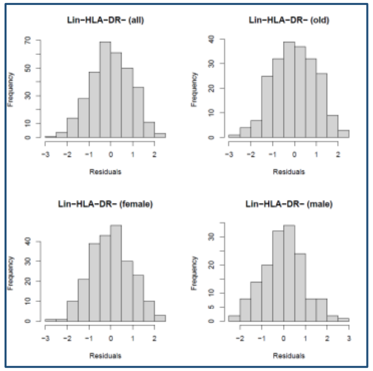

Supplement: Supplementary file 1 [file ijms-25-13655-s001.zip › Deecke_immunePD_PGS_Supplementary_Material_revision2_proof.pdf]
